# Society News for Issue 13(2)

**DOI:** 10.1093/nop/npag010

**Published:** 2026-05-07

**Forthcoming Meetings**

**American Association for Cancer Research (AACR) Annual Meeting**

April 17-22, 2026—San Diego, CA

https://www.aacr.org/meeting/aacr-annual-meeting-2026/

**Annual Meeting of the American Academy of Neurology**

April 18-22, 2026—Chicago, IL

https://www.aan.com/events/annual-meeting

**Brain Tumor Epidemiology Consortium**

May 20-22, 2026—London, UK

https://sites.usc.edu/braintumorcause/btec-conference-2026-london/

**American Society of Clinical Oncology (ASCO) Annual Meeting**

May 29-June 2, 2026—Chicago, IL

https://www.asco.org/annual-meeting

**International Stereotactic Radiosurgery Society Congress**

May 31-June 3, 2026—Sydney, Australia

https://isrscongress.org/

**Asian Society for Neuro-Oncology (ASNO) Annual Meeting**

June 12-14, 2026—Kanazawa, Japan

https://site2.convention.co.jp/asno2026/

**22nd International Symposium on Pediatric Neuro-Oncology (ISPNO)**

June 28-July 1, 2026—Sydney, Australia

https://www.ispno2026.org/

**British Neuro-Oncology Society Annual Meeting (BNOS)**

July 1-3, 2026—Birmingham, UK

https://www.bnos.org.uk/bnos-2026/

**SIOPE Brain Tumour Group Annual Meeting & Educational Course**

September 15-18, 2026—Athens, Greece

https://siope.eu/SIOPE-Brain-Tumour-Group

**European Association of Neuro-Oncology (EANO) Annual Meeting**

September 24-27, 2026—Rome, Italy

https://www.eano.eu/eano2026/

